# Substantial Change Between Repeated Lipoprotein(a) Measurements Is Associated with Residual Cardiovascular Risk in a Real-World Multicenter Cohort

**DOI:** 10.3390/jcm15176527

**Published:** 2026-08-24

**Authors:** Mi-Na Kim, Dongkuk Kim, Soon Jun Hong, Cheol Woong Yu, Seung Yong Shin, Eung Ju Kim, Hyung Joon Joo

**Affiliations:** 1 Division of Cardiology, Department of Internal Medicine, Korea University Anam Hospital, Seoul 02841, Republic of Korea; alsk1229@gmail.com (M.-N.K.); psyche94@hanmail.net (S.J.H.); ycw717@naver.com (C.W.Y.); 2 Korea University Research Institute for Medical Bigdata Science, Korea University, Seoul 02841, Republic of Korea; dongkukkim19@gmail.com; 3 Division of Cardiology, Department of Internal Medicine, Korea University Ansan Hospital, Ansan-si 15355, Republic of Korea; theshin04@naver.com; 4 Division of Cardiology, Department of Internal Medicine, Korea University Guro Hospital, Seoul 08308, Republic of Korea; withnoel@empas.com

**Keywords:** lipoprotein(a), variability, atherosclerotic cardiovascular disease, cardiovascular outcomes, risk stratification, real-world data

## Abstract

**Background/Objectives:** Lipoprotein(a) [Lp(a)] is a genetically determined, largely stable risk factor for atherosclerotic cardiovascular disease, and guidelines recommend at least one measurement during adulthood. Whether intra-individual change in Lp(a) between repeated measurements carries prognostic information beyond static risk categories in routine practice is uncertain. **Methods:** In a multicenter retrospective cohort from three South Korean tertiary hospitals, we studied adults with two Lp(a) measurements at least 90 days apart (2019–2024; *n* = 13,914). High variability was defined as an absolute change > 10 mg/dL combined with a relative change > 25%. The primary outcome was major adverse cardiovascular events (MACE), analyzed with Cox proportional hazards models adjusted for clinical covariates including baseline and follow-up Lp(a) risk categories. **Results:** Despite a strong rank correlation between measurements (Spearman’s ρ = 0.92), 2156 patients (15.5%) showed high variability. MACE occurred more frequently in the high-variability group (6.8% vs. 4.2%, *p* < 0.001), and high variability remained independently associated with MACE (adjusted hazard ratio 1.46, 95% CI 1.18–1.80). The association was consistent across multiple sensitivity analyses, persisted after excluding heart-failure hospitalization from MACE (HR 1.41), and was present for both increases and decreases in Lp(a). Incremental discrimination over a base model was modest, though statistically significant (ΔC-statistic 0.006, 95% CI 0.001–0.012; continuous net reclassification index 0.090, *p* < 0.001). **Conclusions:** Intra-individual change in Lp(a) between two measurements is independently associated with cardiovascular outcomes but provides only modest incremental discrimination. These hypothesis-generating findings require prospective validation before they can inform repeat-testing strategies.

## 1. Introduction

Lipoprotein(a) [Lp(a)] is elevated in approximately 20–25% of the global population and has been shown, independent of traditional risk factors, to be significantly associated with atherosclerotic cardiovascular disease (ASCVD) and calcific aortic stenosis [1,2]. Epidemiologic studies indicate a linear relationship between Lp(a) concentration and ASCVD risk across multiple ethnic groups [1]. Individuals above the 75th percentile of Lp(a) have a higher risk of myocardial infarction (MI) [3], and those above the 90th percentile also show an increased risk of heart failure (HF) [4]. More recently, elevated Lp(a) has also been linked to a greater risk of ischemic stroke [5].

Accordingly, dyslipidemia guidelines from the European Atherosclerosis Society (EAS), the European Society of Cardiology (ESC), and the Canadian Cardiovascular Society (CCS) recommend Lp(a) measurement at least once during adulthood to identify individuals with markedly elevated levels who may benefit from intensified ASCVD risk management [6,7,8]. Because Lp(a) concentrations are largely genetically determined and generally stable over time, routine repeat measurement has not been widely recommended [9]. However, direct evidence supporting long-term intra-individual stability in adults remains limited, and several recent reports have described measurable variation in Lp(a) concentrations between repeated measurements [10]. Whether such variability meaningfully alters cardiovascular risk classification in routine clinical practice remains poorly characterized. We therefore sought to determine whether intra-individual variability in Lp(a) provides prognostic information beyond static Lp(a) categories in a real-world tertiary-care population.

## 2. Materials and Methods

### 2.1. Study Design and Data Sources

This multicenter retrospective cohort analysis used harmonized electronic health records from three tertiary university hospitals in South Korea, standardized under the Observational Medical Outcomes Partnership (OMOP) Common Data Model curated by the OHDSI collaboration [11]. ICD-10 diagnoses, prescriptions, and laboratory results were mapped to OMOP concept identifiers and queried directly from Microsoft SQL Server.

### 2.2. Study Population

Adults with at least two Lp(a) measurements separated by ≥90 days between 1 January 2019 and 13 January 2025 were eligible. For individuals with more than two measurements, the first measurement and the next measurement obtained at least 90 days later were used. The mean interval between the two measurements was 426 ± 236 days (median 378 days, interquartile range 292–516). Of 15,775 individuals initially identified, 1776 were excluded because their follow-up Lp(a) measurement was obtained after 13 January 2024, leaving less than 12 months of potential observation before the end of data collection on 13 January 2025. A further 50 were excluded because a study-defined major adverse cardiovascular event occurred between the first and second Lp(a) measurements (on or before time zero), and 35 because no clinical follow-up was available after the follow-up measurement. The final analytic cohort comprised 13,914 patients.

### 2.3. Serum Lipoprotein(a) Measurement

Lp(a) was measured by an immunoturbidimetric assay (Randox LP3403; Randox Laboratories, Crumlin, UK) on a Beckman Coulter AU5800 analyzer (Beckman Coulter, Inc., Brea, CA, USA) in routine clinical practice [11]. A polyclonal anti-apo(a) antibody and a five-point calibration traceable to WHO/IFCC SRM 2B were applied to minimize isoform bias, with intra- and inter-assay coefficients of variation of 2.4–3.2%. Results were expressed in mg/dL. Lp(a) was categorized per the European Atherosclerosis Society consensus [6] as low risk (<30 mg/dL), intermediate (‘grey zone’, 30 to <50 mg/dL), or high risk (≥50 mg/dL). High Lp(a) variability was defined as an absolute change >10 mg/dL combined with a relative change >25% between the two measurements, the operational dual criterion used in our earlier registry report [11]. The component thresholds were informed by prior repeated-measure studies [12,13]. In this study, ‘variability’ denotes the operationally defined change between two repeated measurements, not variability estimated from multiple serial samples.

### 2.4. Definitions of Covariates and Outcomes

Hypertension, diabetes mellitus, dyslipidemia, and chronic kidney disease were ascertained using standard clinical thresholds [11]: blood pressure ≥ 140/90 mmHg or antihypertensive use; HbA1c ≥ 6.5%, fasting glucose ≥ 126 mg/dL, or antidiabetic use; total cholesterol ≥ 240 mg/dL, low-density lipoprotein cholesterol (LDL-C) ≥ 160 mg/dL, triglycerides ≥ 200 mg/dL, high-density lipoprotein cholesterol (HDL-C) < 40 mg/dL, or lipid-lowering therapy; and CKD-EPI estimated glomerular filtration rate (eGFR) < 60 mL/min/1.73 m^2^. Prior myocardial infarction, stroke, heart failure, liver disease, and thyroid disease were identified through OMOP-CDM concept identifiers.

The primary outcome was the incidence of MACE, defined as a composite of cardiovascular death, myocardial infarction or stroke occurring during follow-up (incident or recurrent), coronary revascularization, or hospitalization for heart failure, starting from the date of the follow-up Lp(a) measurement (time zero). Myocardial infarction during follow-up was defined when a patient was admitted for chest pain, angina, or dyspnea with a rise and/or fall of cardiac biomarkers (creatine kinase-MB and/or cardiac troponin) with at least one value exceeding the 99th-percentile upper reference limit, an operational, electronic health record–based definition aligned with the universal definition of myocardial infarction. Stroke during follow-up was defined when the OMOP-CDM concept ID for stroke was newly recorded, or the brain magnetic resonance imaging report showed acute, subacute, or recent cerebral infarction. Acute HF hospitalization required (1) admission via the emergency department, (2) elevated N-terminal pro–B-type natriuretic peptide (NT-proBNP) or B-type natriuretic peptide (BNP), and (3) administration of intravenous furosemide. NT-proBNP thresholds were >450 pg/mL (<55 years), >900 pg/mL (55–75 years), and >1800 pg/mL (>75 years) [14]; a BNP > 400 pg/mL was an alternative criterion [15].

### 2.5. Statistical Analysis

Categorical variables are summarized as *n* (%) and continuous variables as mean ± SD. Between-group comparisons used Student’s *t*-test or the Mann–Whitney U test for continuous variables (normality assessed by the Shapiro–Wilk test) and the χ^2^ or Fisher’s exact test for proportions. Within-individual concordance between baseline and follow-up Lp(a) was quantified by Spearman’s ρ.

Adjusted hazard ratios (HRs) with 95% confidence intervals (CIs) were estimated using Cox proportional hazards models. Follow-up began at the second (follow-up) Lp(a) measurement (time zero) and continued until the first MACE, the last clinical contact, or 3 years, whichever occurred first. Participants without an event were right-censored at their last documented clinical contact. A 90-day landmark analysis was performed to assess the influence of early events. The prespecified multivariable model included indicator variables for baseline and follow-up Lp(a) risk categories together with the Lp(a) variability group, in addition to age, sex, body mass index, smoking and alcohol use, socioeconomic status, comorbidities (hypertension, diabetes mellitus, dyslipidemia, chronic kidney disease, prior myocardial infarction, prior stroke, and prior heart failure), medication use (antihypertensive, antidiabetic agents, statins, ezetimibe, and PCSK9 inhibitors), and laboratory parameters (eGFR, LDL-C, HDL-C, triglycerides, and glucose). Continuous covariates were entered using clinically interpretable increments (Lp(a) and lipids per 10 mg/dL, and eGFR per 10 mL/min/1.73 m^2^ decrease). Because high-sensitivity C-reactive protein (hs-CRP) was missing in approximately 60% of patients, it was excluded from the primary multivariable model to avoid restricting the analysis to patients with measured hs-CRP; the prespecified model was therefore fitted without backward variable selection, and a complete-case analysis additionally adjusting for hs-CRP, together with a multiple-imputation analysis of the primary-model covariates (multiple imputation by chained equations, 40 imputations) across all 13,914 patients, were performed as sensitivity analyses.

To examine whether the observed variability reflected a methodological artifact rather than a prognostically meaningful signal, several additional analyses were performed. First, patients were classified as having stable, increased, or decreased Lp(a), and MACE risk was compared across these directional groups; an association in both directions would be inconsistent with a purely unidirectional drift but would not exclude regression to the mean or analytical variation. Second, the analytical reference-change value (1.96 × √2 × the assay coefficient of variation) was calculated and reported for context. Using the upper reported assay coefficient of variation of 3.2%, this corresponded to a relative change of 8.9%. In an additional analysis, patients were classified solely according to whether the absolute relative change exceeded this reference-change value, independently of the primary dual-threshold definition. Because our exposure definition (>25% relative change) necessarily exceeds this analytical threshold, this comparison is descriptive and does not, by itself, distinguish biological from analytical variation. Third, the association was examined within each baseline Lp(a) category and after excluding patients within 5 mg/dL of the 30 or 50 mg/dL boundaries to address potential category-boundary artifacts. In addition, to assess the influence of elective revascularization, a composite restricted to cardiovascular death, myocardial infarction, stroke, and heart-failure hospitalization was analyzed.

To assess robustness, multiple sensitivity analyses were performed, including continuous adjustment for baseline and follow-up Lp(a), adjustment for the inter-measurement interval, a 90-day landmark analysis, exclusion of patients with chronic kidney or thyroid disease, and alternative adjustments using time-averaged Lp(a). The incremental predictive value of Lp(a) variability was evaluated by the change in Harrell’s C-statistic, with 95% CIs from 1000 bootstrap resamples, the integrated discrimination improvement (IDI), and the continuous net reclassification index (NRI); overall model fit was compared using the likelihood ratio test. To address potential overadjustment among the mathematically related Lp(a) terms, four nested models—baseline covariates alone and with the baseline Lp(a) category, the follow-up Lp(a) category, or the variability indicator added separately—were compared by Harrell’s C-statistic (Supplementary Table S3). In addition, the cumulative incidence of MACE in patients with stable, increased, or decreased Lp(a) was compared using Kaplan–Meier estimates and the log-rank test. Further details are provided in Supplementary Methods S1 and S2. All analyses were conducted in R (version 4.1.2), with two-sided *p* values < 0.05 considered statistically significant. The sensitivity and robustness analyses were intended as internal consistency checks rather than independent hypothesis tests, and no formal correction for multiplicity was applied.

## 3. Results

### 3.1. Baseline Characteristics

A total of 13,914 patients were analyzed. Baseline and follow-up Lp(a) concentrations were strongly rank-correlated (Spearman’s ρ = 0.92). Nevertheless, marked changes in Lp(a) levels were observed in a subset of patients, and 2156 individuals (15.5%) were classified as having high Lp(a) variability. Baseline characteristics according to Lp(a) variability status are summarized in Table 1.

Patients in the high-variability group had substantially higher Lp(a) concentrations at baseline than those in the low-variability group (44.7 ± 38.1 vs. 23.0 ± 28.9 mg/dL, *p* < 0.001), and Lp(a) concentrations at follow-up remained significantly higher (54.4 ± 41.0 vs. 23.2 ± 28.7 mg/dL, *p* < 0.001). Consistent with this higher Lp(a) burden, patients with high Lp(a) variability exhibited a more adverse clinical profile, including greater age and a higher prevalence of hypertension, diabetes mellitus, dyslipidemia, chronic kidney disease, and prior stroke, as well as greater systemic inflammation and more intensive medical therapy. These findings indicate that high Lp(a) variability is preferentially observed among patients with higher baseline Lp(a) levels and an increased cardiorenal and metabolic risk burden.

### 3.2. Primary Cardiovascular Outcomes

**Figure 1:**
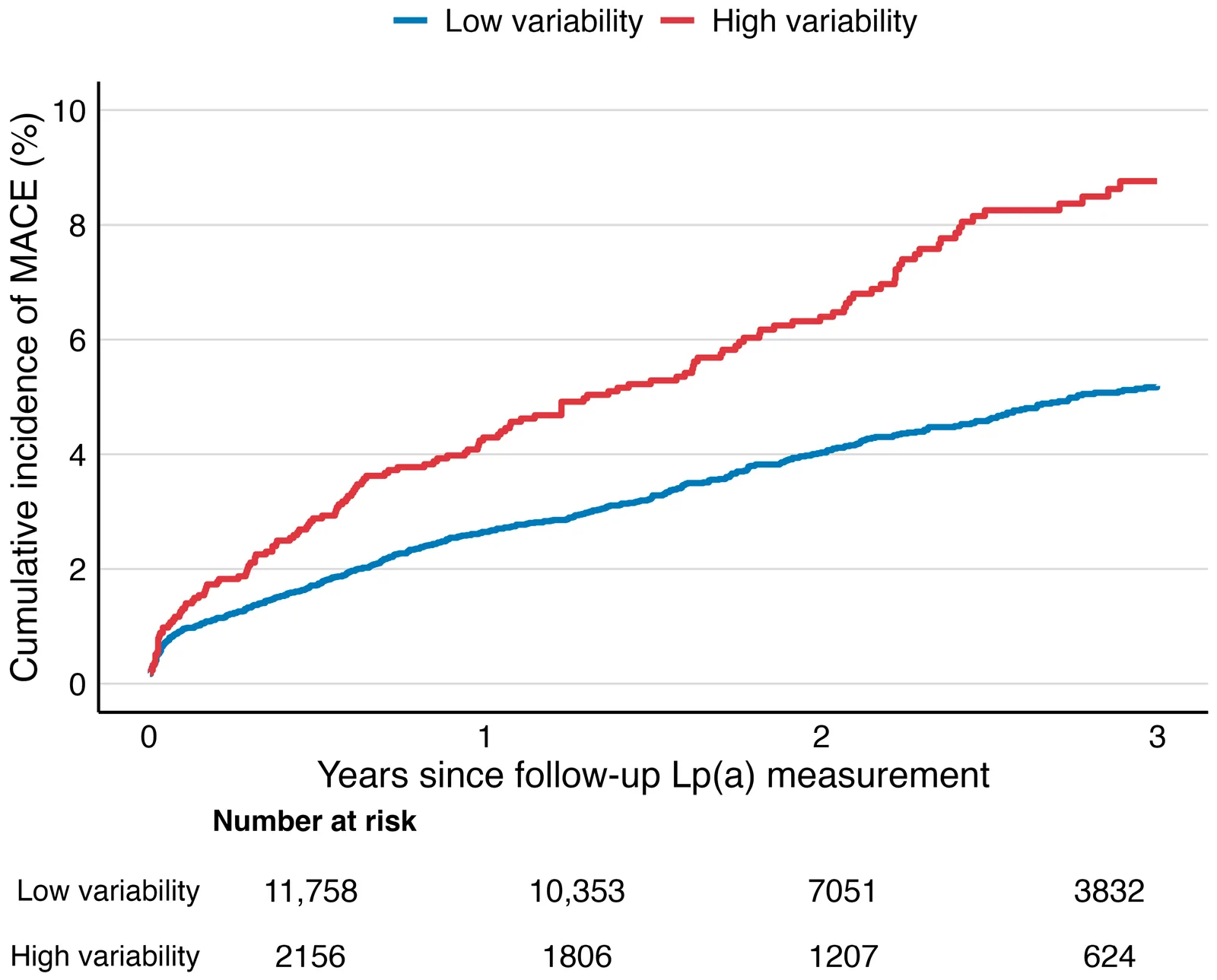
Cumulative incidence of major adverse cardiovascular events (MACE) by Lp(a) variability group, from the date of the follow-up Lp(a) measurement. MACE includes cardiovascular death, myocardial infarction or stroke during follow-up, coronary revascularization, or hospitalization for heart failure. Lp(a), lipoprotein(a).

During follow-up, MACE occurred more frequently in the high-variability group (6.8% vs. 4.2%, log-rank *p* < 0.001, Figure 1). Among individual outcomes, patients with high Lp(a) variability had a greater risk of myocardial infarction during follow-up (1.0% vs. 0.5%, *p* = 0.017) and heart-failure hospitalization (2.4% vs. 1.2%, *p* < 0.001), whereas stroke during follow-up did not differ between groups (0.9% vs. 0.8%, *p* = 0.860). Cardiovascular death was infrequent and similar between groups (6/11,758 [0.1%] vs. 1/2156 [<0.1%], *p* = 1.000). High Lp(a) variability was associated with a higher rate of percutaneous coronary intervention (PCI) (3.8% vs. 2.3%, *p* < 0.001), whereas coronary artery bypass grafting was infrequent and did not differ between groups (Table 2).

Over a mean follow-up of 774 ± 321 days (median 846 days, interquartile range 519–1095) and 29,475 person-years, MACE occurred at 19.6 and 33.6 per 1000 person-years in the low- and high-variability groups, respectively. In the multivariable Cox model adjusted for clinical covariates including baseline and follow-up Lp(a) risk categories (Table 3), high Lp(a) variability remained independently associated with the primary outcome (adjusted HR 1.46, 95% CI 1.18–1.80, *p* < 0.001, N = 12,354 with complete covariate data). Because hs-CRP was missing in approximately 60% of patients, it was excluded from the primary model to avoid restricting the analysis. In the subset with available hs-CRP (N = 5370), the association remained after additional adjustment for hs-CRP (HR 1.70, 95% CI 1.28–2.27). In the same subset, the model without hs-CRP gave a similar estimate (HR 1.73, 95% CI 1.30–2.30), indicating that hs-CRP adjustment had little effect and that the higher estimate in this subset may reflect selective hs-CRP testing or differences in case mix rather than confounding. Multiple imputation of the primary-model covariates across all 13,914 patients gave a concordant estimate (HR 1.46, 95% CI 1.18–1.81). Collinearity diagnostics did not indicate a material effect on the variability estimate (variance inflation factor 1.2), although the mutually adjusted baseline and follow-up Lp(a) category coefficients (generalized variance inflation factor 1.5 each) are not interpretable as independent dose–response estimates. When the variability indicator was instead added to the baseline covariate model without the two Lp(a) category terms, it remained associated with MACE (HR 1.43, 95% CI 1.19–1.72), indicating that the association was not an artifact of adjusting for the mathematically related Lp(a) terms in a single model; each Lp(a) term added only a small increment to discrimination (Harrell’s C 0.704 for the baseline covariates alone, 0.706 with the variability indicator, and 0.709 in the full model; Supplementary Table S3). Because the number of covariates was large relative to the number of events, a reduced model restricted to major risk factors gave a consistent result (HR 1.52, 95% CI 1.23–1.88). The PCSK9-inhibitor estimate, based on only 58 exposed patients, was imprecise and should be interpreted with caution.

To assess whether the composite was driven by elective revascularization, the analysis was repeated using a composite restricted to cardiovascular death, myocardial infarction, stroke, and heart-failure hospitalization. This composite occurred in 3.9% versus 2.3% of patients, and high variability remained independently associated after adjustment (HR 1.45, 95% CI 1.08–1.93). The association also persisted when heart-failure hospitalization was instead excluded from the primary composite (HR 1.41, 95% CI 1.11–1.79).

### 3.3. Robustness and Incremental Predictive Value

**Figure 2:**
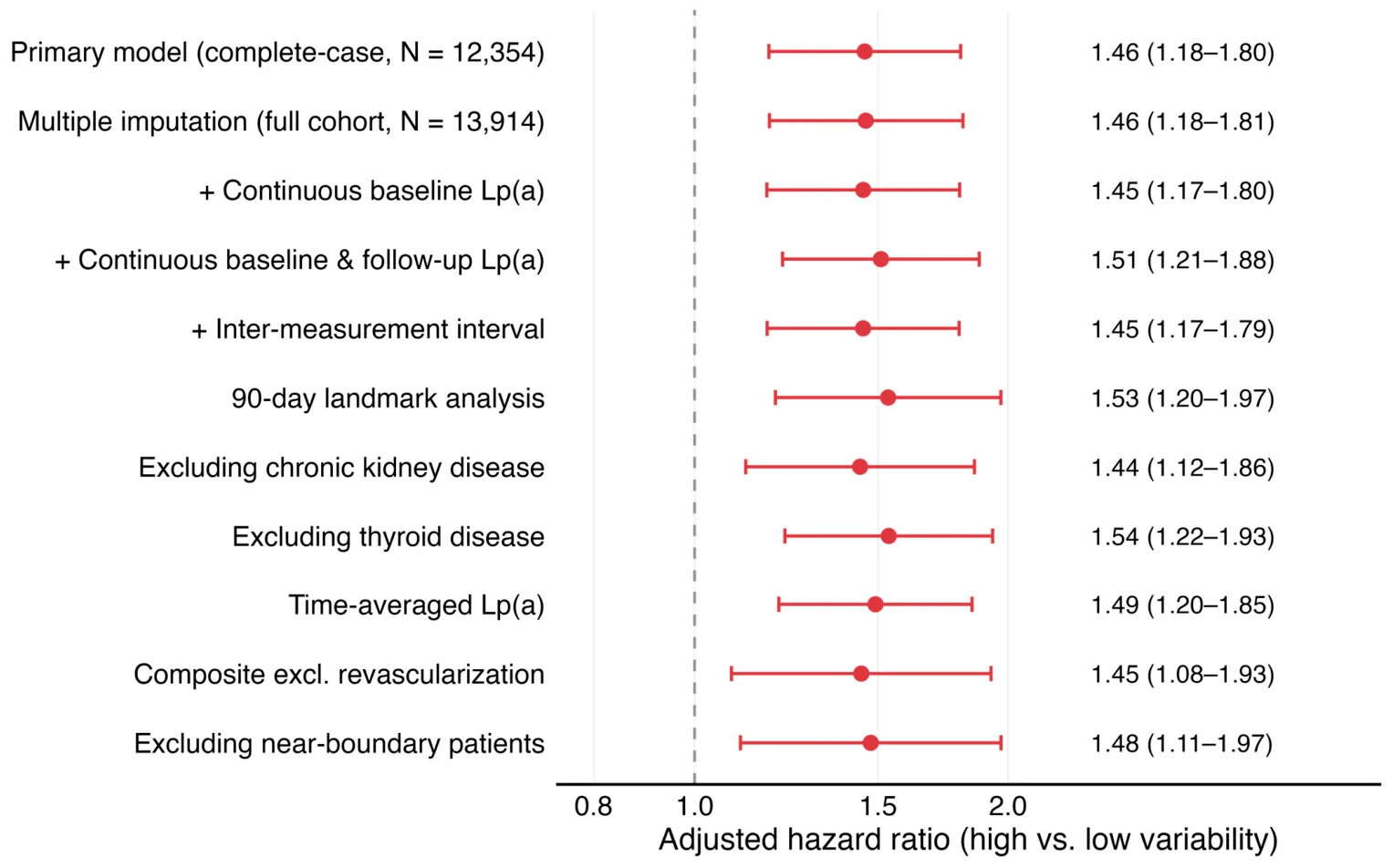
Sensitivity and robustness analyses for the association between Lp(a) variability and MACE. Forest plot of adjusted hazard ratios with 95% confidence intervals comparing high vs. low Lp(a) variability across sensitivity models and additional robustness analyses, including a composite excluding coronary revascularization and exclusion of patients near category boundaries. Detailed values are provided in Supplementary Table S1. CI, confidence interval; HR, hazard ratio; Lp(a), lipoprotein(a). The vertical dashed line indicates the null value (HR = 1.0).

**Figure 3:**
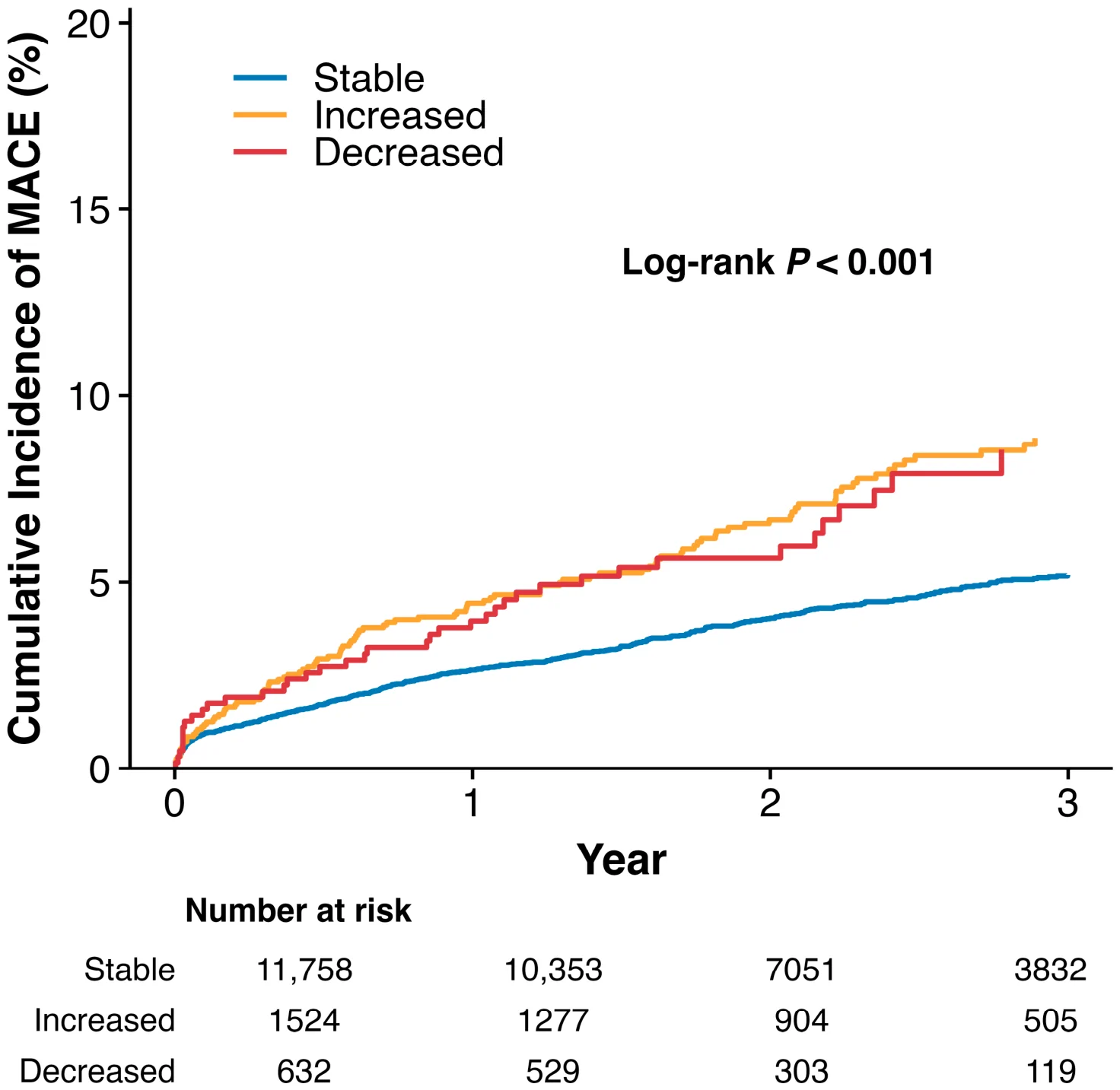
Cumulative incidence of major adverse cardiovascular events (MACE) according to the direction of Lp(a) change. Kaplan–Meier estimates for patients with stable (*n* = 11,758), increased (*n* = 1524), and decreased (*n* = 632) Lp(a) between the two measurements. MACE, major adverse cardiovascular events.

The association between Lp(a) variability and MACE was consistent across sensitivity and robustness analyses (Figure 2 and Supplementary Table S1). Across the sensitivity analyses displayed in Figure 2, adjusted hazard ratios ranged from 1.44 to 1.54, including continuous adjustment for baseline and follow-up Lp(a), the inter-measurement interval, a 90-day landmark analysis, exclusion of chronic kidney or thyroid disease, and time-averaged Lp(a). The alternative-outcome and directional analyses were consistent with the primary result, whereas subgroup estimates varied, with an association in the low- and high-risk categories but not in the intermediate-risk category (Supplementary Table S1). Three analyses addressed whether the signal reflects a methodological artifact. First, within the primary complete-case cohort, both increases (*n* = 1390, 108 events, HR 1.39, 95% CI 1.08–1.81) and decreases (*n* = 568, 38 events, HR 1.59, 95% CI 1.11–2.27) in Lp(a) were associated with MACE relative to stable values, a pattern inconsistent with a purely unidirectional drift, although this does not exclude regression to the mean or analytical variation. In a Kaplan–Meier analysis of the full cohort, the 3-year cumulative incidence of MACE was higher in both the increased and decreased groups than in the stable group (8.9% and 8.6% versus 5.2%; overall log-rank *p* < 0.001; Figure 3), whereas the increased and decreased groups did not differ (log-rank *p* = 0.75), supporting that the magnitude rather than the direction of change carried the prognostic signal. Second, because the study definition (>25% relative change) necessarily exceeds the analytical 95% reference-change value derived from the assay coefficient of variation (8.9% relative change), we report this comparison descriptively rather than as evidence excluding analytical variation. Third, the association was preserved within the baseline low- and high-risk Lp(a) categories (HR 1.44 and 1.56, respectively) and after excluding patients near the 30 and 50 mg/dL category boundaries (HR 1.48, 95% CI 1.11–1.97), arguing against a boundary artifact. The variability-by-baseline-category interaction was not significant (*p* = 0.65). However, this test was based on few events in the intermediate-risk stratum and is underpowered rather than definitive evidence of homogeneity.

The incremental discrimination afforded by Lp(a) variability over a base risk model was modest and, being derived in the same cohort, reflects apparent (in-sample) discrimination (Table 4). Addition of Lp(a) variability increased Harrell’s C-statistic from 0.678 to 0.683 (ΔC-statistic = 0.006, bootstrap 95% CI 0.001–0.012, likelihood ratio test *p* < 0.001). The integrated discrimination improvement (0.002, *p* < 0.001) and continuous net reclassification index (0.090, *p* < 0.001) were statistically significant but small in magnitude, indicating that Lp(a) variability refines rather than replaces existing risk assessment.

### 3.4. Lp(a) Risk Category and Variability

Of the 13,914 patients, 10,254 (73.7%) were in the low-risk Lp(a) category, 1598 (11.5%) in the intermediate-risk category, and 2062 (14.8%) in the high-risk category at baseline. The distribution of Lp(a) risk categories differed substantially between variability groups at both index and follow-up (Table 5). Most patients in the low- and high-risk categories remained in their baseline category during follow-up, whereas 46.9% of those initially classified as intermediate risk were reclassified, with 24.4% transitioning to the high-risk category (Table 6). Across categories, MACE occurred more frequently among patients classified as high-risk at both baseline and follow-up (Supplementary Table S2). However, in an exploratory analysis confined to the baseline intermediate-risk category (full stratum 1598 patients with 65 events; adjusted complete-case analysis, 1401 patients with 64 events), high variability was not associated with MACE (HR 1.06, 95% CI 0.56–2.00). Given the small number of events, this analysis was underpowered, and the variability-by-category interaction (*p* = 0.65) should not be read as definitive. Thus, although the intermediate-risk category showed the greatest category instability over time, variability did not independently predict events within this subgroup; the prognostic association was instead observed within the low- and high-risk categories (Supplementary Table S1).

## 4. Discussion

In this large real-world multicenter cohort, longitudinal change in Lp(a) was independently associated with increased residual cardiovascular risk, beyond both baseline and follow-up Lp(a) categories. The association was consistent across the covariate-adjustment and cohort-restriction analyses (Figure 2). At the population level, the incremental discrimination added by Lp(a) variability over a base risk model was modest (ΔC-statistic = 0.006, 95% CI 0.001–0.012). Lp(a) variability is therefore best regarded as a complement to established risk factors and static Lp(a) risk stratification, not a replacement, and these findings should be interpreted as a prognostic association rather than evidence that variability is a directly actionable target.

Although Lp(a) is widely viewed as a temporally stable biomarker, our findings indicate that longitudinal change identifies a subgroup of patients with a higher cardiovascular event rate, even after accounting for baseline and follow-up Lp(a) risk categories. Because the composite was partly influenced by revascularization, which is susceptible to indication bias in tertiary care, we confirmed that the association persisted for a composite excluding coronary revascularization.

Although the clinical implications of intra-individual Lp(a) variability remain limited [16,17], accumulating evidence suggests that meaningful longitudinal variation can occur in certain clinical contexts [18,19]. Cao et al. reported that greater Lp(a) variability was associated with an increased risk of MACE among patients with familial hypercholesterolemia [17]. Our findings are concordant with this observation and extend it to a real-world tertiary-care population. In contrast, Trinder et al. reported no significant association between changes in Lp(a) concentrations over time and coronary artery disease (*p* = 0.98) [12], and Ghouse et al. reported that Lp(a) concentrations remained highly stable over time [20]. These discrepancies are probably explained by differences in the definition of Lp(a) variability and in the underlying study populations: both Trinder et al. and Ghouse et al. used large, population-based biobank cohorts of predominantly healthy participants, whereas our hospital-based cohort was enriched for patients with established cardiovascular disease or multiple risk factors and intensive lipid-lowering therapy [12,20]. The present analysis is based on the same multicenter Lp(a) registry as our earlier report, which examined the clinical and biochemical predictors of high Lp(a) variability and the associated risk-factor profiles and category reclassification in a smaller sample (*n* = 5305) [11]. The two studies share the data source and the operational definition of high variability (>10 mg/dL and >25% change) but address different questions: the earlier work characterized who develops variability and how it reclassifies static risk categories, whereas the present study, in an expanded cohort (*n* = 13,914) with longitudinal follow-up, evaluates whether variability independently predicts incident MACE and quantifies its incremental prognostic value beyond baseline and follow-up Lp(a) categories. The outcome models, incremental-value and robustness analyses, and clinical conclusions are new, and no tables are reproduced from the earlier report.

The mechanisms underlying the relationship between Lp(a) variability and adverse outcomes remain incompletely understood. Lp(a) plasma concentrations may exhibit context-dependent fluctuations in response to systemic inflammation, oxidative stress, or hormonal and metabolic perturbations [21]; increased inflammation might transiently alter hepatic apo(a) synthesis or Lp(a) catabolism [22,23]. The higher hs-CRP levels and greater prevalence of chronic kidney disease observed among patients with high variability are consistent with a contribution of systemic inflammation and impaired renal clearance. In keeping with a shared inflammatory pathway, adjustment for hs-CRP produced only minimal attenuation of the association within the subset in whom it was measured (HR 1.73 without versus 1.70 with hs-CRP). The larger estimate in this complete-case subset than in the primary model (HR 1.46) may reflect selective hs-CRP testing, differences in case mix, or effect heterogeneity rather than confounding, since the same subset already yielded HR 1.73 before hs-CRP was added and a multiple-imputation analysis across the full cohort closely reproduced the primary estimate (HR 1.46). Lp(a) comprises an LDL-like apoB-100–containing particle covalently linked to apo(a), which confers distinct proatherogenic and prothrombotic properties [24], and fluctuations could reflect variations in oxidized phospholipids, endothelial dysfunction, and vascular inflammation that promote plaque progression and instability [25,26]. Statins and ezetimibe exert minimal or modestly increasing effects on Lp(a), whereas PCSK9 inhibitors reduce it [27,28,29]; the higher use of these agents among patients with greater variability may reflect both confounding by indication and treatment-related changes in Lp(a). Variability was most pronounced among individuals in the intermediate-risk range, a biologically heterogeneous group in whom mid-range concentrations may be more sensitive to inflammatory, metabolic, or renal modulation. This mid-range predominance may also partly reflect the variability definition, which combines an absolute (>10 mg/dL) and a relative (>25%) threshold that are jointly easier to satisfy at intermediate Lp(a) concentrations. However, because variability did not independently predict events within this subgroup, any mechanistic interpretation of the intermediate-risk range remains speculative and unproven. Both increases and decreases in Lp(a) were associated with MACE. This bidirectionality is inconsistent with a purely unidirectional drift but cannot, by itself, distinguish biological change from analytical or pre-analytical variation or regression to the mean.

This study has several limitations. First, the retrospective observational design precludes causal inference, and residual confounding cannot be fully excluded despite comprehensive multivariable adjustment. In particular, the positive associations of ezetimibe and PCSK9 inhibitors with MACE most likely reflect confounding by indication and should not be interpreted as treatment effects, although a reduced model omitting these treatment covariates revealed a consistent estimate for Lp(a) variability (Section 3.2). Second, only two Lp(a) measurements per individual were available; two measurements capture a single difference rather than true variability, and person-level indices such as the coefficient of variation or average real variability could not be computed. Nonetheless, the bidirectional association is inconsistent with a purely unidirectional drift, although it cannot exclude analytical or pre-analytical variation or regression to the mean. Because the Lp(a) risk-category boundaries (30 and 50 mg/dL) are dichotomous, small absolute changes near these thresholds might shift categorical classification; however, the association persisted within baseline categories and after excluding near-boundary patients. Third, all laboratories used the same immunoturbidimetric assay with ongoing quality control, yet residual analytical and pre-analytical variability cannot be entirely excluded; results were consistent after adjustment for the inter-measurement interval and in a 90-day landmark analysis. Fourth, the cohort was drawn from tertiary centers enriched for high-risk, intensively treated patients in whom repeat Lp(a) was typically obtained for clinical reasons. The resulting selection and indication bias means that the question of who warrants repeat testing in primary prevention cannot be addressed by this design, and reverse causation cannot be excluded. Nevertheless, the association persisted for a composite excluding coronary revascularization. In addition, because inclusion required survival, event-free, to a second measurement and follow-up began at that measurement, events occurring between the two draws were excluded by design. This immortal-time and survivor bias is inherent to the primary design and is only partially addressed by the 90-day landmark analysis. Fifth, genetic determinants of Lp(a), including apo(a) isoform size, were unavailable. Sixth, the incremental discrimination added by Lp(a) variability was modest, so its value is best understood as refining rather than replacing existing risk stratification. Finally, as the study was conducted in tertiary hospital cohorts in South Korea, generalizability to community-based settings or other ethnic populations warrants further investigation.

Our findings should be interpreted as a prognostic association rather than a basis for changing testing practice. The incremental discrimination added by Lp(a) variability over a base risk model was modest, the cohort was enriched for high-risk, intensively treated patients, and variability did not independently predict events within the intermediate-risk category highlighted by earlier work. We therefore refrain from recommending repeat Lp(a) measurement for any specific subgroup. Whether serial Lp(a) assessment improves risk stratification or outcomes—and whether it is relevant for selecting or monitoring patients as Lp(a)-lowering therapies such as pelacarsen and olpasiran advance toward clinical use [30,31]—is a hypothesis that requires prospective evaluation.

## 5. Conclusions

In a real-world multicenter cohort, intra-individual change in Lp(a) between two measurements was independently associated with cardiovascular outcomes but added only modest incremental discrimination. These hypothesis-generating findings warrant prospective validation before any change to Lp(a) testing strategy.

## Figures and Tables

**Table 1:** Baseline characteristics of the study population by Lp(a) variability.

Characteristic	Low Lp(a) Variability (*n* = 11,758)	High Lp(a) Variability (*n* = 2156)	*p*-Value
Lp(a), baseline, mg/dL	23.0 ± 28.9	44.7 ± 38.1	<0.001
Lp(a), follow-up, mg/dL	23.2 ± 28.7	54.4 ± 41.0	<0.001
Age, years	64.6 ± 12.6	66.2 ± 12.1	<0.001
Sex, male, *n* (%)	6932 (59.0)	1242 (57.6)	0.252
Body mass index, kg/m^2^	24.8 ± 3.6	24.4 ± 3.8	<0.001
Smoking, *n* (%)	5125 (43.6)	965 (44.8)	0.325
Alcohol, *n* (%)	5782 (49.2)	1071 (49.7)	0.686
Hypertension, *n* (%)	9421 (80.1)	1831 (84.9)	<0.001
Diabetes, *n* (%)	5488 (46.7)	1131 (52.5)	<0.001
Dyslipidemia, *n* (%)	10,583 (90.0)	2012 (93.3)	<0.001
Chronic kidney disease, *n* (%)	1353 (11.5)	386 (17.9)	<0.001
Myocardial infarction, *n* (%)	1432 (12.2)	289 (13.4)	0.120
Stroke, *n* (%)	2306 (19.6)	491 (22.8)	0.001
Heart failure, *n* (%)	1042 (8.9)	215 (10.0)	0.107
eGFR, mL/min/1.73 m^2^	87.5 ± 19.5	84.2 ± 22.9	<0.001
Total cholesterol, mg/dL	153.1 ± 38.4	152.4 ± 41.9	0.553
LDL-C, mg/dL	77.5 ± 29.3	77.0 ± 32.0	0.519
HDL-C, mg/dL	51.0 ± 12.8	49.9 ± 13.5	<0.001
Triglyceride, mg/dL	122.0 ± 70.2	123.6 ± 77.3	0.375
Glucose, mg/dL	111.8 ± 27.7	113.9 ± 37.0	0.014
hs-CRP, mg/L	1.22 ± 1.61	1.52 ± 1.89	<0.001
Antihypertensive, *n* (%)	8075 (68.7)	1607 (74.5)	<0.001
Antidiabetic, *n* (%)	3655 (31.1)	743 (34.5)	0.002
Statin, *n* (%)	5616 (47.8)	1127 (52.3)	<0.001
Ezetimibe, *n* (%)	4666 (39.7)	957 (44.4)	<0.001
PCSK9 inhibitor, *n* (%)	35 (0.3)	23 (1.1)	<0.001

Continuous variables are mean ± standard deviation; categorical variables are *n* (%). eGFR, estimated glomerular filtration rate; HDL-C, high-density lipoprotein cholesterol; hs-CRP, high-sensitivity C-reactive protein; LDL-C, low-density lipoprotein cholesterol; Lp(a), lipoprotein(a); PCSK9, proprotein convertase subtilisin/kexin type 9.

**Table 2:** Cardiovascular outcomes by Lp(a) variability group.

Outcome	Low Lp(a) Variability (*n* = 11,758)	High Lp(a) Variability (*n* = 2156)	*p*-Value
MACE	492 (4.2)	147 (6.8)	<0.001
Cardiovascular death	6 (0.1)	1 (<0.1)	1.000
MI during follow-up	61 (0.5)	21 (1.0)	0.017
Stroke during follow-up	96 (0.8)	19 (0.9)	0.860
HF hospitalization	137 (1.2)	51 (2.4)	<0.001
PCI	274 (2.3)	83 (3.8)	<0.001
CABG	5 (<0.1)	1 (<0.1)	1.000

Values are *n* (%). CABG, coronary artery bypass grafting; MACE, major adverse cardiovascular events; MI, myocardial infarction; PCI, percutaneous coronary intervention.

**Table 3:** Multivariable Cox proportional hazards model for major adverse cardiovascular events.

Predictor	HR	95% CI	*p*-Value
Lp(a) variability (high vs. low)	1.46	1.18–1.80	<0.001
Age, per 1-year increase	1.019	1.010–1.028	<0.001
Male sex	1.194	0.998–1.428	0.053
Smoking	1.050	0.860–1.282	0.631
Alcohol	1.597	1.298–1.964	<0.001
Low socioeconomic status	1.564	1.237–1.979	<0.001
Body mass index, per 1 kg/m^2^ increase	0.975	0.952–0.998	0.036
Hypertension	1.311	0.903–1.903	0.155
Diabetes	1.156	0.937–1.425	0.176
Dyslipidemia	1.131	0.708–1.809	0.606
Chronic kidney disease	1.092	0.814–1.467	0.557
Prior myocardial infarction	1.555	1.267–1.907	<0.001
Prior stroke	0.763	0.628–0.928	0.007
Prior heart failure	1.095	0.870–1.379	0.440
eGFR, per 10 mL/min/1.73 m^2^ decrease	1.078	1.015–1.144	0.015
LDL-C, per 10 mg/dL increase	1.033	1.004–1.064	0.028
HDL-C, per 10 mg/dL increase	0.872	0.809–0.939	<0.001
Triglyceride, per 10 mg/dL increase	0.997	0.985–1.009	0.614
Glucose, per 10 mg/dL increase	1.024	1.008–1.041	0.004
Antihypertensive	1.452	1.092–1.930	0.010
Antidiabetic	1.293	1.055–1.585	0.013
Statin	1.067	0.892–1.277	0.478
Ezetimibe	1.434	1.216–1.693	<0.001
PCSK9 inhibitor	2.241	1.147–4.377	0.018
Baseline Lp(a), grey zone vs. low	0.708	0.511–0.980	0.038
Baseline Lp(a), high vs. low	0.834	0.554–1.255	0.384
Follow-up Lp(a), grey zone vs. low	1.015	0.756–1.363	0.919
Follow-up Lp(a), high vs. low	1.251	0.827–1.892	0.289

Prespecified multivariable model restricted to complete cases for all covariates (N = 12,354, 633 events). The difference from the analytic cohort (13,914) reflects missing covariate data, mainly body mass index. CI, confidence interval; eGFR, estimated glomerular filtration rate; HDL-C, high-density lipoprotein cholesterol; HR, hazard ratio; LDL-C, low-density lipoprotein cholesterol; Lp(a), lipoprotein(a); PCSK9, proprotein convertase subtilisin/kexin type 9.

**Table 4:** Incremental discrimination of Lp(a) variability for major adverse cardiovascular events (apparent, in-sample).

Metric	Base Model	Base Model + Lp(a) Variability	Change (95% CI)	*p*-Value
Harrell’s C-statistic	0.678	0.683	0.006 (0.001–0.012)	—
Likelihood ratio test	—	—	—	<0.001
Integrated discrimination improvement	—	—	0.002 (0.001–0.006)	<0.001
Continuous net reclassification index	—	—	0.090 (0.031–0.129)	<0.001

The base model comprised age, sex, baseline and follow-up Lp(a) risk categories, hypertension, diabetes, prior myocardial infarction, prior stroke, statin use, eGFR, and LDL-C. The second model added the binary Lp(a) variability indicator. Complete-case N = 13,643 (635 events). This base model uses fewer covariates than Table 3, so its complete-case sample is larger. ΔC was calculated from unrounded C-statistics, and its 95% CI from 1000 bootstrap resamples. CI, confidence interval.

**Table 5:** Distribution of Lp(a) risk categories at index and follow-up by variability group.

	Low Lp(a) Variability (*n* = 11,758)	High Lp(a) Variability (*n* = 2156)	*p*-Value
Lp(a) risk at index			<0.001
Low, *n* (%)	9348 (79.5)	906 (42.0)	
Intermediate, *n* (%)	1003 (8.5)	595 (27.6)	
High, *n* (%)	1407 (12.0)	655 (30.4)	
Lp(a) risk at follow-up			<0.001
Low, *n* (%)	9280 (78.9)	649 (30.1)	
Intermediate, *n* (%)	1089 (9.3)	609 (28.2)	
High, *n* (%)	1389 (11.8)	898 (41.7)	

*p*-values from chi-squared test. Lp(a), lipoprotein(a).

**Table 6:** Transition of Lp(a) risk categories from baseline to follow-up.

Baseline Lp(a) Risk	Follow-Up Low	Follow-Up Intermediate	Follow-Up High
Low (*n* = 10,254)	9542 (93.1)	636 (6.2)	76 (0.7)
Intermediate (*n* = 1598)	359 (22.5)	849 (53.1)	390 (24.4)
High (*n* = 2062)	28 (1.4)	213 (10.3)	1821 (88.3)

Values are *n* (%). Lp(a), lipoprotein(a). Low, <30 mg/dL; Intermediate, 30 to <50 mg/dL; High, ≥50 mg/dL.

## Data Availability

The de-identified data are not publicly available owing to privacy restrictions.

## Supplementary Materials

The following supporting information can be downloaded at https://www.mdpi.com/article/10.3390/jcm15176527/s1, Supplementary Methods S1: Cox proportional hazards modelling; Supplementary Methods S2: Incremental predictive value of Lp(a) variability; Table S1: Sensitivity and robustness analyses for the association between Lp(a) variability and major adverse cardiovascular events; Table S2: Cardiovascular events by baseline and follow-up Lp(a) risk category. Table S3: Comparison of Harrell’s C-statistics across nested multivariable models.
