# Supplementary material for: Substantial Change Between Repeated Lipoprotein(a) Measurements Is Associated with Residual Cardiovascular Risk in a Real-World Multicenter Cohort
Source: J Clin Med. 2026 Aug 24;15(17):6527. doi: 10.3390/jcm15176527 (PMC13566148; doi:10.3390/jcm15176527)
Supplement: Supplementary file 1 [file jcm-15-06527-s001.zip › jcm-4451224-supplementary.pdf]

Supplementary Information

Substantial Change Between Repeated Lipoprotein(a) Measurements Is Associated with Residual Cardiovascular Risk in a Real-World Multicenter Cohort

Contents

Supplementary Methods S1. Cox proportional hazards modelling

Supplementary Methods S2. Incremental predictive value of Lp(a) variability

Table S1. Sensitivity and robustness analyses for the association between Lp(a) variability and major adverse cardiovascular events

Table S2. Cardiovascular events by baseline and follow-up Lp(a) risk category

Table S3. Comparison of Harrell's C-statistics across nested multivariable models

Supplementary Methods S1. Cox proportional hazards modelling

Adjusted hazard ratios (HRs) with 95% confidence intervals were estimated using Cox proportional hazards regression. The prespecified multivariable model (covariates as listed in the main-text Methods) was fitted in the primary complete-case sample (N = 12,354, 633 events) without backward selection. Follow-up was censored at the last clinical contact or 3 years, whichever came first. High-sensitivity C-reactive protein was excluded from this model owing to approximately 60% missingness and was instead examined in a complete-case sensitivity analysis (N = 5,370). To assess the potential impact of complete-case exclusion under a missing-at-random assumption, missing data in the primary-model covariates (predominantly body mass index) were addressed by multiple imputation by chained equations (predictive mean matching, 40 imputations) across all 13,914 patients, with estimates combined using Rubin's rules. The pooled hazard ratio for high versus low Lp(a) variability was 1.46 (95% CI, 1.18–1.81), matching the complete-case estimate. The model is reported in Table 3 of the main manuscript.

Sensitivity and robustness analyses, summarized in Table S1 and Figure 2, comprised continuous adjustments for baseline and follow-up Lp(a), the inter-measurement interval, a 90-day landmark analysis, exclusion of chronic kidney and/or thyroid disease, time-averaged Lp(a), a directional (increase or decrease vs. stable) analysis, within-category and near-boundary analyses, and a hard composite excluding revascularization. Analyses used R version 4.1.2 (survival package); two-sided $p < 0.05$ was considered significant.

Supplementary Methods S2. Incremental predictive value of Lp(a) variability

Two nested Cox models were compared: a base model (age, sex, baseline and follow-up Lp(a) risk categories, hypertension, diabetes, prior myocardial infarction, prior stroke, statin use, estimated glomerular filtration rate, and LDL-cholesterol; hs-CRP excluded to allow analysis in the larger complete-case sample (N = 13,643)) and a full model adding the binary high-variability indicator. Discrimination was assessed by Harrell's C-statistic, with the change (ΔC) and its 95% confidence interval from 1,000 bootstrap replicates; nested models were compared by the likelihood ratio test; and reclassification was quantified by the continuous net reclassification index and integrated discrimination improvement at 3 years (survIDINRI package). Results are presented in Table 4 of the main manuscript. Modest improvements were anticipated a priori given the high baseline performance of conventional risk factors and Lp(a) levels.

Table S1. Sensitivity and robustness analyses for the association between Lp(a) variability and major adverse cardiovascular events.

Analysis	Adjusted HR	95% CI	p-value
Alternative covariate adjustment			
Primary model (complete-case, N = 12,354)	1.46	1.18–1.80	<0.001
Additionally adjusted for continuous baseline Lp(a)	1.45	1.17–1.80	<0.001
Additionally adjusted for continuous baseline and follow-up Lp(a)	1.51	1.21–1.88	<0.001
Additionally adjusted for inter-measurement interval	1.45	1.17–1.79	<0.001
Adjusted for time-averaged Lp(a)	1.49	1.20–1.85	<0.001
Complete-case analysis, additionally adjusted for hs-CRP	1.70	1.28–2.27	<0.001
Multiple imputation of primary-model covariates (N = 13,914; 639 events)	1.46	1.18–1.81	<0.001
Restriction of the study population			
90-day landmark analysis	1.53	1.20–1.97	<0.001
Excluding patients with chronic kidney disease	1.44	1.12–1.86	0.005
Excluding patients with thyroid disease	1.54	1.22–1.93	<0.001
Excluding patients with chronic kidney or thyroid disease	1.47	1.12–1.94	0.006
Excluding patients near the 30 or 50 mg/dL boundaries	1.48	1.11–1.97	0.008
Alternative outcome definition			
Composite excluding coronary revascularization	1.45	1.08–1.93	0.012
Composite excluding heart-failure hospitalization	1.41	1.11–1.79	0.005
Subgroup by baseline Lp(a) category			
Low-risk category	1.44	1.04–2.00	0.028
Intermediate (grey-zone) category	1.06	0.56–2.00	0.864
High-risk category	1.56	1.06–2.28	0.023
Direction of Lp(a) change (vs. stable)			
Increased Lp(a)	1.39	1.08–1.81	0.012
Decreased Lp(a)	1.59	1.11–2.27	0.011
Alternative exposure classification			
Reference-change-value threshold only	1.16	0.94–1.42	0.160

Adjusted HR compares high versus low Lp(a) variability unless otherwise specified. Models are adjusted for the primary Cox covariates. Analyses are grouped by type: alternative covariate adjustment (same outcome, with analytic sample sizes varying where indicated), restriction of the study population, alternative outcome definition, subgroup by baseline category, direction of change, and alternative exposure classification. For the direction-of-change analysis, analytic group sizes (events) were stable 10,396 (487), increased 1,390 (108), and decreased 568 (38), with hazard ratios estimated from a single model referenced to the stable group. The variability-by-baseline-category interaction was not significant (likelihood ratio test p = 0.65). CI, confidence interval; HR, hazard ratio; hs-CRP, high-sensitivity C-reactive protein; Lp(a), lipoprotein(a). The reference-change-value-only classification, defined solely by exceeding the analytical reference-change value, did not show statistically significant evidence of an association with MACE.

Table S2. Cardiovascular events by baseline and follow-up Lp(a) risk category.

Outcome	Baseline Low (n=10,254)	Baseline Inter. (n=1,598)	Baseline High (n=2,062)	p	Follow-up Low (n=9,929)	Follow-up Inter. (n=1,698)	Follow-up High (n=2,287)	p
MACE	456 (4.4)	65 (4.1)	118 (5.7)	0.023	423 (4.3)	80 (4.7)	136 (5.9)	0.002
CV death	6 (0.1)	0 (0.0)	1 (<0.1)	1.000	6 (0.1)	0 (0.0)	1 (<0.1)	0.845
MI during follow-up	57 (0.6)	12 (0.8)	13 (0.6)	0.616	56 (0.6)	10 (0.6)	16 (0.7)	0.747
Stroke during follow-up	88 (0.9)	6 (0.4)	21 (1.0)	0.081	79 (0.8)	12 (0.7)	24 (1.0)	0.407
HF hospitalization	128 (1.2)	19 (1.2)	41 (2.0)	0.025	114 (1.1)	24 (1.4)	50 (2.2)	0.001
PCI	248 (2.4)	42 (2.6)	67 (3.2)	0.092	237 (2.4)	45 (2.7)	75 (3.3)	0.050
CABG	4 (<0.1)	1 (0.1)	1 (<0.1)	0.646	3 (<0.1)	2 (0.1)	1 (<0.1)	0.160

Values are n (%). p-values are from the χ^2 test, except for cardiovascular death and coronary artery bypass grafting, for which Fisher's exact test was used owing to sparse cells. CABG, coronary artery bypass grafting; CV, cardiovascular; HF, heart failure; Inter., intermediate; MACE, major adverse cardiovascular events; MI, myocardial infarction; PCI, percutaneous coronary intervention.

Table S3. Comparison of Harrell's C-statistics across nested multivariable models.

Model	Harrell's C (95% CI)	ΔC vs baseline	Lp(a) variability HR (95% CI)
Baseline covariates only	0.704 (0.684–0.724)	Reference	—
+ Baseline Lp(a) category	0.706 (0.686–0.725)	+0.002	—
+ Follow-up Lp(a) category	0.705 (0.685–0.725)	+0.001	—
+ Lp(a) variability	0.706 (0.687–0.726)	+0.002	1.43 (1.19–1.72)
Full model (both categories + variability)	0.709 (0.689–0.729)	+0.005	1.46 (1.18–1.80)

All models were fitted in the primary complete-case sample (N = 12,354; 633 events) using the full multivariable covariate set. Baseline covariates comprise all primary-model covariates except the two Lp(a) terms. C, Harrell's concordance statistic; CI, confidence interval; HR, hazard ratio; Lp(a), lipoprotein(a); MACE, major adverse cardiovascular events.
